# Supplementary material for: Dihydrothiazolo ring-fused 2-pyridone antimicrobial compounds treat Streptococcus pyogenes skin and soft tissue infection
Source: Sci Adv. 2024 Aug 2;10(31):eadn7979. doi: 10.1126/sciadv.adn7979 (PMC11296344; doi:10.1126/sciadv.adn7979)
Supplement: Supplementary file 1 — Figs. S1 to S6 Tables S1 to S3 [file sciadv.adn7979_sm.pdf]

Science Advances
AAAS

Supplementary Materials for

Dihydrothiazolo ring-fused 2-pyridone antimicrobial compounds treat *Streptococcus pyogenes* skin and soft tissue infection

Zongsen Zou *et al.*

Corresponding author: Fredrik Almqvist, fredrik.almqvist@umu.se; Scott J. Hultgren, hultgren@wustl.edu; Michael G. Caparon, caparon@wustl.edu

Sci. Adv. **10**, eadn7979 (2024)
DOI: 10.1126/sciadv.adn7979

The PDF file includes:

Figs. S1 to S6
Tables S1 to S3

Other Supplementary Material for this manuscript includes the following:

Data S1

SUPPLEMENTARY FIGURES

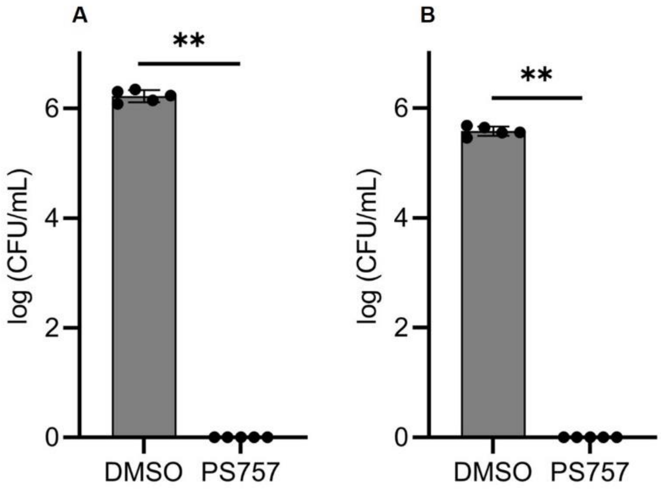

Fig. S1. GmPcide PS757 demonstrates robust bactericidal activity against the exponential- and stationary-phase of *S. pyogenes* HSC5 cells. (A) Exponential-phase (7 hours post inoculum) *S. pyogenes* HSC5 cells treated under bactericidal (20 µM) concentration of PS757 for 12 hours were observed with > 6.0 logCFU reduction. **(B)** Stationary-phase (14 hours post inoculum) *S. pyogenes* HSC5 cells treated under bactericidal (20 µM) concentration of PS757 for 12 hours were observed with > 5.0 logCFU reduction. Statistics were performed with Mann-Whitney U test. $P \leq 0.05$ is considered as statistically significant. *$P \leq 0.05$, **$P < 0.01$, ***$P < 0.001$, ****$P < 0.0001$, ns indicates not significant.

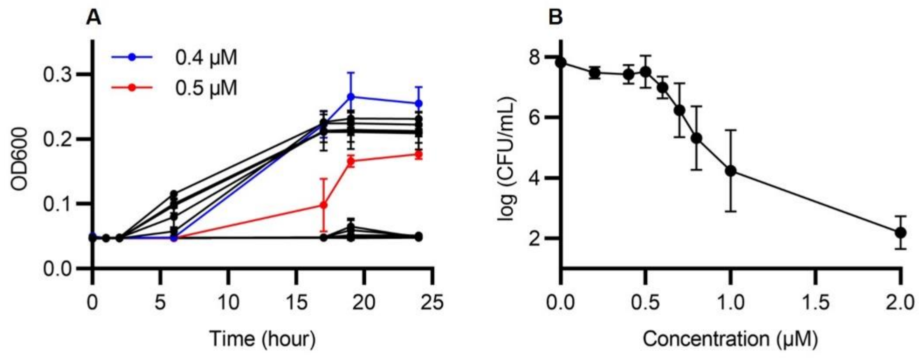

Fig. S2. Sublethal concentration of GmPcide PS757 against *S. pyogenes* HSC5 was determined in microplate assay using C medium by measuring both **(A)** OD600 and **(B)** CFU, which identified 0.4 μM as the sublethal concentration of PS757 against *S. pyogenes* HSC5.

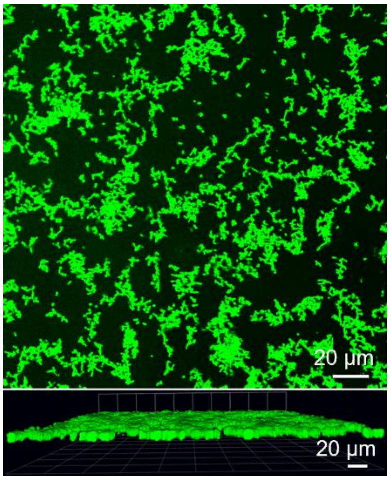

Fig. S3. Immunofluorescent confocal microscopy characterization of *S. pyogenes* HSC5 biofilm at 24 hrs post-inoculation. A dense biofilm with an approximate thickness of 9.5 µm was observed.

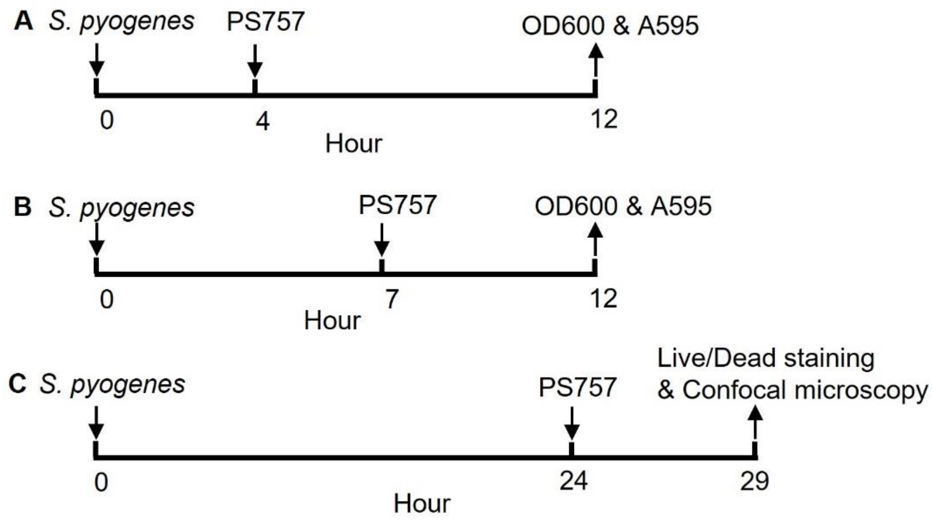

Fig. S4. GmPcide PS757 treatment to different phases of *S. pyogenes* biofilm. (A) GmPcide PS757 treatment to *S. pyogenes* HSC5 biofilm at 4 hrs during initiation phase. **(B)** GmPcide PS757 treatment to *S. pyogenes* HSC5 biofilm 7 hrs during maturing development. **(C)** GmPcide PS757 treatment to mature *S. pyogenes* HSC5 biofilm at 24 hrs.

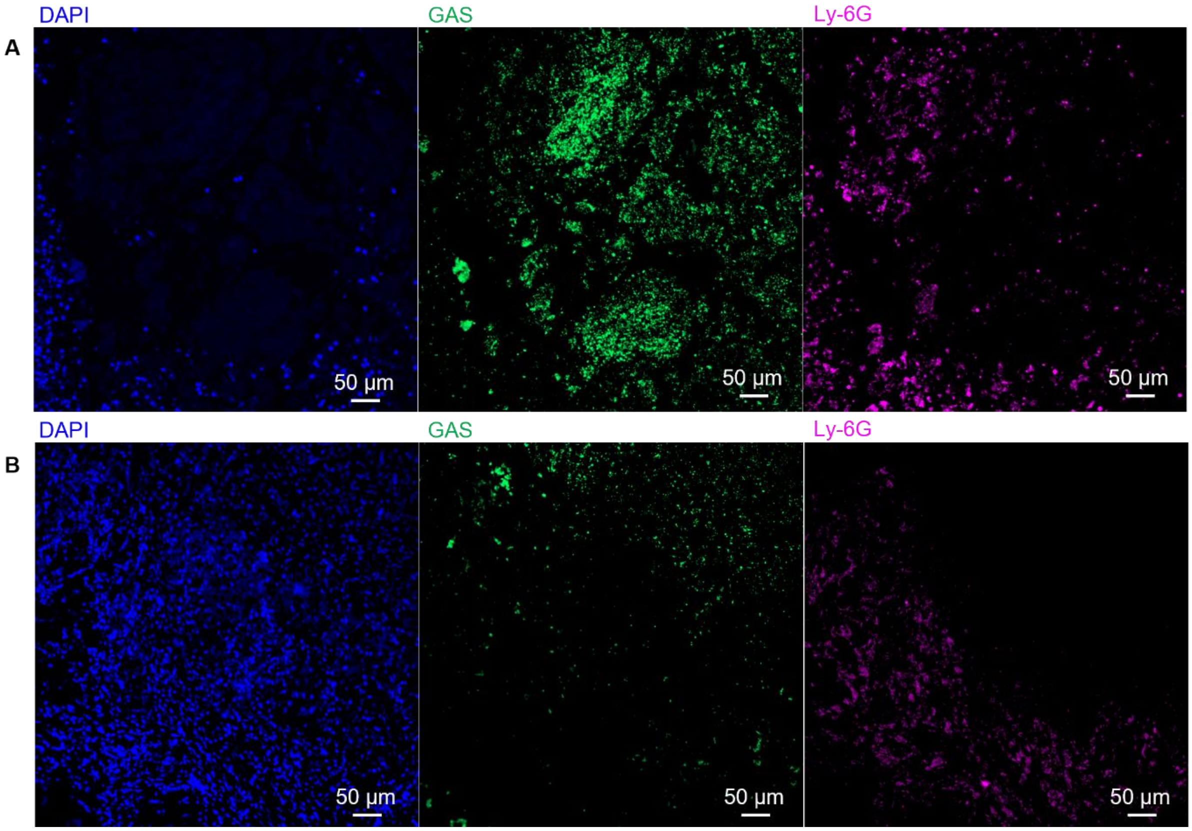

Fig. S5. Immunofluorescence microscopy characterizations of thigh tissue samples from vehicle-treated (DMSO) (A) and PS757-treated (B) mice. Host cell nuclei, *S. pyogenes* HSC5, and polymorphonuclear neutrophils were stained with DAPI, GAS, and Ly-6G antibodies, respectively.

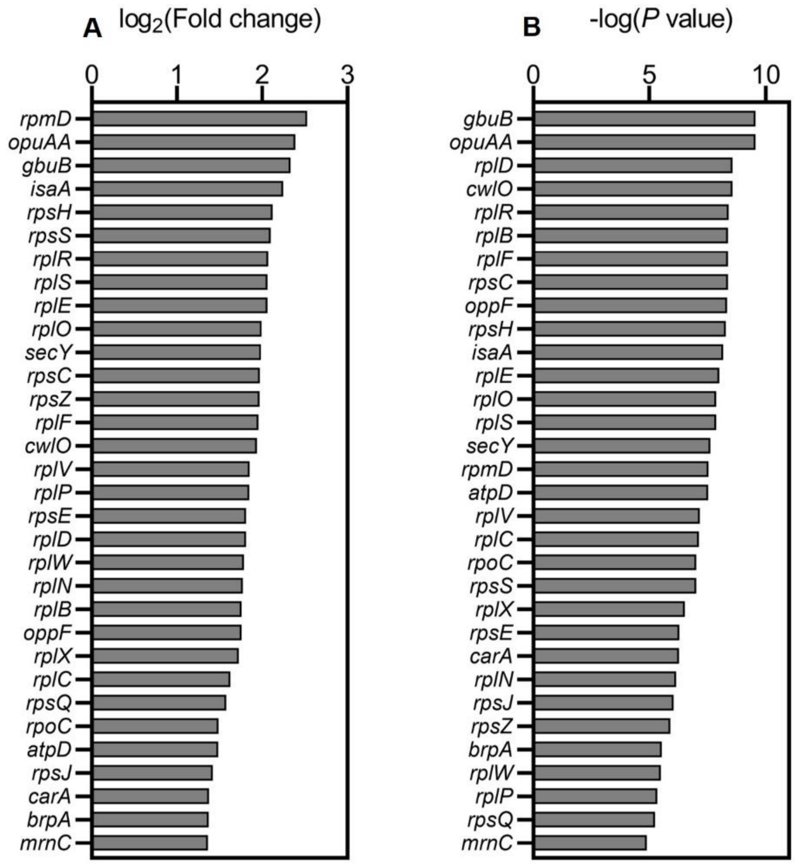

Fig. S6. Differentially expressed genes (DEGs) induced by PS757 treatment were identified as $\log_2$(FC) > 0.5 and P < 0.05 in the comparative RNA-seq analysis. Among DEGs, two more stringent selection criteria, $|\log_2(\mathrm{FC})|$ **(A)** and -log(P) **(B)** > 99% confidence intervals (CI) upper limits were applied to select the most up-regulated group of genes, which identified 32 most up-regulated genes featuring the involvement of two ribosomal protein-associated pathways, Rpl and Rps.

SUPPLEMENTARY TABLES

Table S1. MIC and MBC concentrations of four standard-of-care antibiotics against *S. pyogenes* HSC5

Strain	Antibiotic	Concentration (µM)	
		**MIC**	**MBC**
S. pyogenes HSC5	Penicillin	0.09	0.35
	Cefotaxime	0.13	0.52
	Vancomycin	0.17	0.34
	Azithromycin	0.32	5.10

Table S2. Antimicrobial synergy of GmPcide PS757 with four standard-of-care antibiotics examined by checkerboard method and E test.

Strain	Combined treatment		MIC (μM)		FICI
			Alone	Combined	
S. pyogenes HSC5	PS757 + Penicillin	PS757	0.78	0.39	0.75
		Penicillin	0.09	0.02	
	PS757 + Cefotaxime	PS757	0.78	0.39	0.75
		Cefotaxime	0.13	0.03	
	PS757 + Vancomycin	PS757	0.78	0.39	1.50
		Vancomycin	0.17	0.17	
	PS757 + Azithromycin	PS757	0.78	0.39	1.50
		Azithromycin	0.32	0.32	

Table S3. The list of most down- and up-regulate S. pyogenes genes induced by sublethal PS757 treatment identified by comparative transcriptomic analysis.

**Differentially expressed levels (DELs)**	**Differentially expressed genes (DEGs)**
Most down-regulated	*emm5*, *malQ*, *udp*, *malX*, *nupX*, *ycjP*, *arlR*, *NA*, *NA*
Most up-regulated	*rpmD*, *opuAA*, *gbuB*, *isaA*, *rpsH*, *rpsS*, *rplR*, *rplS*, *rplE*, *rplO*, *secY*, *rpsC*, *rpsZ*, *rplF*, *cwlO*, *rplV*, *rplP*, *rpsE*, *rplD*, *rplW*, *rplN*, *rplB*, *oppF*, *rplX*, *rplC*, *rpsQ*, *rpoC*, *atpD*, *rpsJ*, *carA*, *brpA*, *mrnC*, *NA*
